# A school-based physical activity promotion intervention in children: rationale and study protocol for the PREVIENE Project

**DOI:** 10.1186/s12889-017-4788-4

**Published:** 2017-09-26

**Authors:** Pablo Tercedor, Emilio Villa-González, Manuel Ávila-García, Carolina Díaz-Piedra, Alejandro Martínez-Baena, Alberto Soriano-Maldonado, Isaac José Pérez-López, Inmaculada García-Rodríguez, Sandra Mandic, Juan Palomares-Cuadros, Víctor Segura-Jiménez, Francisco Javier Huertas-Delgado

**Affiliations:** 1 PA-HELP “Physical Activity for HEalth Promotion” Research Group, Granada, Spain; 2 Department of Physical Education and Sport, Faculty of Sport Sciences, University of Granada, Granada, Spain; 3 PROFITH “PROmoting FITness and Health through physical activity” Research Group, Granada, Spain; 4 “Mind, Brain, and Behavior” Research Center, University of Granada, Granada, Spain; 5 “Physical Activity and Pedagogy” Research Unit, Department of Physical Education and Sport, School of Sport Sciences, University of Valencia, Valencia, Spain; 6 “SPORT” Research Group, Department of Education, Faculty of Education Sciences, University of Almería, Almería, Spain; 7 “Physical Education and Social Transformation” Research Group, Granada, Spain; 8 Active Living Laboratory, School of Physical Education, Sport and Exercise Sciences, University of Otago, Dunedin, New Zealand; 9 “Curricular Design, Development and Innovation in the Area of Physical Education Didactics” Research Group, Department of Physical Education and Sport, International University of La Rioja, La Rioja, Spain; 10 Department of Physical Education, School of Education Sciences, University of Cádiz, Cádiz, Spain; 11 Teaching School La Inmaculada, University of Granada, Granada, Spain

## Abstract

**Background:**

The lack of physical activity and increasing time spent in sedentary behaviours during childhood place importance on developing low cost, easy-toimplement school-based interventions to increase physical activity among children. The PREVIENE Project will evaluate the effectiveness of five innovative, simple, and feasible interventions (active commuting to/from school, active Physical Education lessons, active school recess, sleep health promotion, and an integrated program incorporating all 4 interventions) to improve physical activity, fitness, anthropometry, sleep health, academic achievement, and health-related quality of life in primary school children.

**Methods:**

A total of 300 children (grade 3; 8-9 years of age) from six schools in Granada (Spain) will be enrolled in one of the 8-week interventions (one intervention per school; 50 children per school) or a control group (no intervention school; 50 children). Outcomes will include physical activity (measured by accelerometry), physical fitness (assessed using the ALPHA fitness battery), and anthropometry (height, weight and waist circumference). Furthermore, they will include sleep health (measured by accelerometers, a sleep diary, and sleep health questionnaires), academic achievement (grades from the official school’s records), and health-related quality of life (child and parental questionnaires). To assess the effectiveness of the different interventions on objectively measured PA and the other outcomes, the generalized linear model will be used.

**Discussion:**

The PREVIENE Project will provide the information about the effectiveness and implementation of different school-based interventions for physical activity promotion in primary school children.

## Background

Opportunities for children and youth to be physically active have declined in many countries in recent decades due to environmental factors, parental rules, and school policies [1]. Since the strong link between health and education was recognized worldwide [2, 3], public health professionals as well as researchers have identified schools as a strategic place to promote physical activity (PA) [3–6]. In the last decade, schools in the USA and in some European countries have reduced the time devoted to Physical Education (PE) in the school curriculum (in Spain, for example, primary and secondary schools devote 2 h/week to PE) [7]. Results from eight European countries showed that a low percentage of children met the minimum recommended of 60 min of moderate-to vigorous physical activity per day (MVPA) (30.4% in Spain) [8]. Since PA behaviors are developed early in life and may persist throughout childhood and adolescence [9, 10], adequate PA level in childhood may also be essential for the prevention of obesity and chronic diseases in later life [11]. In children, regular PA is associated with reduced rates of obesity [12], improved academic and cognitive achievement [13], better sleep health [14], and improved health-related quality of life [15–17]*.* A reasonable way to assist school-age children to increase their PA levels is to help them take every opportunity to be active throughout the day [18]. Active commuting to school [19], PE lessons, and active school recess [20] could provide children with opportunities to engage in PA during school days.

Children who actively commute to/from school had 24 additional minutes of MVPA per day compared to those who did not [21]. Previous intervention studies in primary school children reported inconsistent results, such as increased rates of cycling and walking to school [22], increased rates of cycling to school only [23], and no effect on rates of cycling to school [24]. Thus, further studies need to examine the effect of an intervention program based on active commuting to/from school. Initiatives such as Safe Routes to School [25, 26], the Walking School Bus [27], the Walk to School program [28], and the School Travel Plan program [29] have been implemented to increase children’s walking and bicycling to/from school with successful results in some cases. However, a systematic review by Chillón et al. [30] concluded that more research with higher quality study designs and measures was needed to identify the most successful strategies for increasing the frequency of active commuting to/from school.

PE lessons are an ideal setting to improve children’s fundamental movement skills, increase PA and physical fitness, and improve health [7, 31, 32]. To achieve these benefits children should be involved in MVPA during at least 50% of the PE lesson time [33]. The majority of previous interventions designed to increase children’s MVPA in PE fall into one of the two following categories: a) interventions that use teaching strategies (e.g. CATCH, SPARK, M-SPAN, Move it Grove it) [31, 34–36], and b) interventions that focus on fitness [33]. The interventions based on teaching strategies showed an increase in MVPA during PE lessons. On the other hand, studies addressing fitness focused on increasing the time of PE lessons [37], which is not possible without a change in the Spanish official curriculum. For example, results from the CATCH intervention increased MVPA during PE lessons by 12% to meet the current recommendation for children’s PA during PE lessons [38]. Other intervention programs found an increase in PA levels during the school day when introducing active breaks in the ordinary lessons [39]. In general, school-based PA interventions focusing mainly on changes in PE lessons could increase PA by 5 to 45 min/dayin children and adolescents [10]. The Government of Spain is currently promoting the participation of key stakeholders (e.g. parents, teachers, and other child educators) to help increase PA levels in children and adolescents [40]. The Spanish Ministry of Health, Social Services, and Equality and the Ministry of Education, Culture, and Sport have developed the so-called “Active Didactic Units” [41] aiming at increasing MVPA during PE lessons. This intervention includes two sets of eight active PE lessons specifically developed for third grade primary school children. It is freely available to all primary schools in Spain.

School recesses provide opportunities to practice motor skills [1], and might contribute to up to 40% of children’s recommended daily PA [42, 43]. School playgrounds are effective settings for implementing school-based interventions to increase PA during school recess [10, 44]. However, there are different ways to increase PA during recess. The use of pedometers to report the number of steps in recess may be effective to increase PA in children [45]. Other ways to increase PA during recess include modifying the schoolyard, using activity cards, using cones to mark activity zones, or increasing the amount of the available playground equipment [46].

MVPA also correlates with sleep duration in children [14, 47] and adolescents [48]. To achieve the recommended9 to 12 h of sleep within a 24 h period [49], it is reasonable to start modifying factors that disturb sleep, including limiting sedentary time [50]. Of particular interest is the relationship established between sleep duration at night and PA/sedentary time during the following day, as their combined effect might increase cardio-metabolic risk markers in childhood [11]. However, there was no increase in sleep duration in a children PA intervention conducted over 1 year [51]. It still remains to be examined if school-based PA interventions could improve sleep duration in primary school children when sleep is objectively measured.

Different interventions including the promotion of active commuting to/from school, PE lessons, school recess interventions, and sleep behavior interventions can be performed in school-based settings. However, no previous study compared the effectiveness of those interventions on increasing PA levels in school-age children. The interventions included in the PREVIENE Project have been designed following the conceptual framework for a comprehensive school-based PA intervention [52]. Thus, the PREVIENE Project aims to examine the effectiveness of five 8-weekschool-based interventions (active commuting to/from school, active PE lessons, active school recess, sleep health promotion, and an integrated intervention with all four components) and compare them with the control group. The final aim of the PREVIENE Project is to improve PA, fitness, anthropometry, sleep health, academic achievement, and health-related quality of life in primary school children.

## Methods/Design

### Study design

**Fig. 1:**
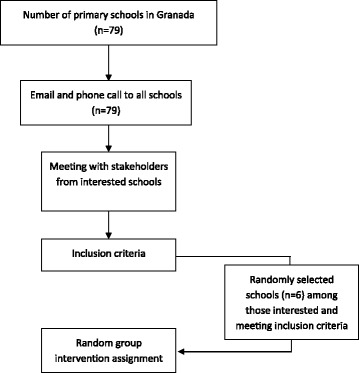
School recruitment diagram for the PREVIENE Study

The PREVIENE Project will use a quasi-experimental approach with a convenience sample size of 300 children (grade 3, 8-9 years old) from six primary schools in Granada (Spain) (2 classes per school × 6 schools = 12classes in total). With an average class size of 25 children and an expected recruitment rate of 90%, two classes will be selected in each participating school. All 79 primary schools in Granada will be invited to participate in this study. The schools will initially be contacted by email followed by a phone call. The research team will arrange a meeting with the schools interested in participating and their staff (principal, physical education teacher, and other relevant teachers). At this meeting, the researchers will explain the main objective of this study and the inclusion criteria for schools’ participation: 1) at least 2 classes of grade 3 children, 2) an average class size of at least 25 children. Once the schools that satisfy the inclusion criteria are determined and they express their willingness to participate, a total of six schools will be randomly selected (Fig. 1).

The Regional Ministry of Education will endorse the participation of each selected school in the study. At the beginning of the study, families of all children in the selected classes will receive an invitation to an initial meeting at school to receive information about the study. Both children and parents will be encouraged to participate in the study. Parents will sign an informed consent which includes both parents’ and their children’s participation. The study protocol has been approved by the University of Granada Human Research Ethics Committee (Reference: 57/CEIH/2015).

**Fig. 2:**
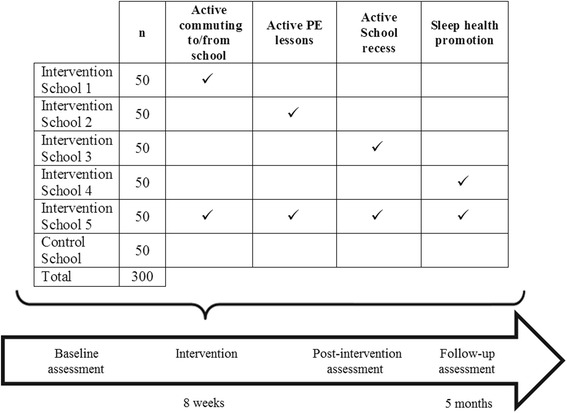
The PREVIENE Project study design

Figure 2 summarizes the study design. Five schools will be assigned an intervention randomly. Four of them will apply a single intervention (*n* = 50 children per intervention/school), and one school will implement the integrated program (all four interventions simultaneously; n = 50 children). The sixth school will serve as a control school and will not receive any intervention (n = 50 children). In schools assigned the active PE lesson and integrated intervention, the standard PE lessons will be replaced with the Active Didactics Unit. In the rest of the schools, children will continue with the usual distribution of PE sessions according to the National Education Program in Spain (i.e. 45 min sessions twice per week).

### Sample size calculations

Based on previously published findings [10], a minimum sample size of 40 children per intervention is required to detect changes across groups in MVPA (the primary outcome) of 10 min/day (SD 13) with a power of 80%, and an α of 0.05. Therefore, a total of 240 participants (40 per intervention × 6 groups) are needed. To account for the potential loss to follow-up of up to 25%, a total of 300 children will be recruited (50 children per intervention).

### Interventions

An outline of the school-based interventions and a summary of intervention-specific activities is presented in Table 1. Considering that school-based PA interventions should be realistic, adapted to the school timetable, and focused on promoting PA opportunities at school, the following four 8-week school-based interventions programs were chosen for this study: 1) active commuting to/from school, 2) active PE lessons, 3) active school recess, and 4) sleep health promotion. In addition, a simultaneous implementation of all four interventions will be examined in one of the intervention schools.

**Table 1:** Interventions and examples of activities

Intervention programs	Implemented by	Examples of activities
Active Commuting to/from School	Teachers	Activities in the school neighborhood to improve children’s autonomy and abilities • Walking in the neighborhood focusing on facilitators they perceive.
	Teachers	Reinforcement of children’s knowledge about active commuting to school. • Working in class through photographs to determine the best appropriate material for active commuting (e.g. proper footwear).
	Parents	Messages to the families with adviceand some of the outcomes of being active • “If you let your children actively commute to school they will become more autonomous”.
Active PE lessons	Teachers	Apply two active educationalunits (8 lessons per unit) replacing the traditional PE lessons. • Different ways to take attendance (i.e. using student as the person responsible of this task) • Selectthe best activity with the available material (i.e. determine the activity based on the number of soccer balls, if the number is low try to use some collective games to ensure the engagement of all children in the class)
Active School Recess	Teachers	• Preparing the playground by offering adequate spaces and games. • Developing games and teaching children to play in recess time (i.e. sport games, traditional games) • Placing a sheet in the classroom with some reminders about being active in recess time (i.e. using a poster board encouraging playing one of the selected games with some images)
Sleep Health Promotion	Teachers	Informative sessions to analyze the best habits prior to sleep time: • Turning off all devices an hour prior to bedtime.
	Parents	
	Teachers	Teaching children Jacobson’s progressive relaxation technique [57].

Researchers carefully designed three of the four interventions: active commuting to/from school, active school recess, and sleep health promotion. The active PE lessons intervention was designed by the Government of Spain [41]. Details of each intervention will be finalized after receiving teachers’ feedback from each participating school. Researchers will train participating teachers in the procedures for applying the assigned intervention at their school. Researchers will also train teachers in promoting behavioral changes in children using the Stages of Change model [53]. Active PE lessons and school recess interventions will be implemented by teachers only. Researchers will help teachers implement the remaining interventions.

#### Active commuting to/from school

This intervention will include a range of school-, family-, and community-based activities [30]. These activities will be focused on children and their families following the ecological model proposed by Sallis et al. [54], targeting mainly individual factors such as children’s perceptions (safety perception on the way to school) and attitudes (independence or motivation to walk). A total of six 1-h activities will be conducted in the classroom and two activities in the school neighborhood. The designed is based on previous literature [23]. Taken together, these activities will promote active commuting to/from school and emphasize its benefits using phrases such as “If you ride a bike for 1,200 km, the average number of sick days will be reduced by one”. Moreover, supporting information will be sent to families on four occasions during the intervention to encourage families to use active modes of commuting to/from school. The supporting information will consist in sending WhatsApp messages in the form of text or images with advice. The aim is to explain the most important ideas related to active commuting and the benefits of active commuting in academic achievements and children’s mental and physical health.

Finally, throughout the intervention period, teachers will ask children about their mode of transport to school of that day twice a week by raising their hand. They will try to encourage possible changes in mode of commuting as motivational strategy by using positive reinforcement (for instance, when children accompany each other on the way to school). The objective of this reinforcement is to remind children to actively commute to and from school.

#### Active PE lessons

This intervention was developed by the Spanish Ministry of Health, Social Services, and Equality and the Ministry of Education, Culture, and Sport [41] with the aim to increase the amount of children’s PA during PE lessons in primary schools. At the time of this study, any school in Spain could choose to adopt this program. This intervention includes two sets of eight active PE lessons specifically developed for third grade primary school children. These lessons will replace the original PE lessons in schools assigned to conduct the Active PE lesson intervention and the integrated intervention. Additionally, this intervention will provide methodological advice to increase the PA time during PE lessons (i.e. different ways to take attendance or deciding on the most suitable activity given the availability of resources).

#### Active school recess

This intervention was designed based on previous research [1, 43, 44, 55]. The teacher will prepare the school playground offering adequate space and games to encourage children to be active. A sheet placed on the wall as a reminder will help teachers remind children to participate and motivate them. On this sheet, each child will write the activity completed during the school recess every day during the intervention period.

#### Sleep health promotion

This intervention will aim to raise awareness of the importance of having a good sleep quality at night and to teach healthy sleep behaviors that will contribute to improving sleep hygiene. As a part of this intervention, eight activities will be carried out at home and at school. During the first activity, parents and children will attend a general talk about sleep and health, and will sign a contract for a “healthy sleep at home”. Signing “the contract” will enable participants to have an active role in the sleep intervention. At home, with the help of their parents, children will complete a diary where they will keep a record of their activities prior to going to bed and after waking up in the morning. The objective of this activity is to strengthen the importance of a routine before going to sleep and its benefits on the adequate sleep behavior. Parents will be given a manual to help children use an adequate sleep routine and reinforce children’s achievements. At school, several teachers assisted by the researchers will implement the activities. The first classroom-based activity will be based on the educational program “I have a dream” (Spanish adaptation of the SimplyHealthy@Schools International Program; Philips Ibérica, S.A., Madrid, Spain). The remaining classroom-based activities will include a group art project with questions and answers about sleep, children’s calculations of their own sleep parameters from the sleep log data. They will form groups and discuss about the sleep diary completed at home and other sleep issues, as well as strategies to achieve the commitments included in the “signed contract”. In addition, the teacher will introduce the concept of relaxation and its benefits at bedtime, and will teach an abbreviated version of the Jacobson’s progressive relaxation technique [56] every morning after the recess.

### Outcome measures and measurement procedures

Children will be assessed at baseline, immediately after the 8-week intervention and 5 months after the intervention using the measurement procedures outlined in Table 2.

**Table 2:** Summary of outcome measures and measurement procedures

Outcome measures	Measurement procedures
Sociodemographic characteristics and health status	Parent questionnaire
Physical activity	Accelerometer (Actigraph wGT3X-BT; 7 consecutive days, 24 h/day): • Physical activity (light, moderate, vigorous, and moderate-to-vigorous PA) • Sedentary time • Sleep (total sleep time, sleep onset latency, sleep efficiency, and wake after sleep onset)
Active commuting to/from school	Child questionnaire
Physical fitness	The ALPHA Fitness Battery: • Handgrip strength • Standing long jump test • 10-m shuttle run tests • 20-m shuttle run test
Anthropometry	Height, weight, and waist circumference
Sleep health	Accelerometer (Actigraph wGT3X-BT; 7 consecutive days, 24 h/day): total sleep time, sleep onset latency, sleep efficiency, and wake after sleep onset. Children sleep log filled out in collaboration with their parents Sleep Knowledge and Hygiene children questionnaire Pediatric Sleep Questionnaire Pediatric Daytime Sleepiness Scale
Academic achievement	Grades from the official school records for Natural Sciences, Social Sciences, Spanish language, Foreign language (English), Music, Arts, and PE Average grade point from the official school records
Health-related quality of life	KINDL-R questionnaire for children KINDL-R questionnaire for parents

#### Sociodemographic characteristics and health status

Parents will complete a questionnaire about the child’s sociodemographic characteristics including date of birth and gender, household factors, family’s socioeconomic characteristics (family income, parental education, and parental employment status), and the child’s health status (reporting medical conditions and medications, if any).

#### Physical activity

PA (light, moderate, vigorous, and moderate-to-vigorous), sedentary time, and sleep parameters (total sleep time, sleep onset latency, sleep efficiency, and wake after sleep onset) will be measured using a tri-axial accelerometer (Actigraph wGT3X-BT, Pensacola, FL, USA) on 7 consecutive days, 24 h/day. Children will be instructed to wear an accelerometer attached to the non-dominant wrist [44, 45]. Teachers, parents, and children will be instructed in the care of the device. Children will take it off only during water-based activities. Children will also complete a log to record the time when they take the accelerometer off.

#### Commuting to school behavior

Children will self-report their mode of commuting to and from school using a paper-based questionnaire and using two sets of questions with two items each: “How do you usually travel to/from school?” and “How do you commute to/from school each day of the week?” [58]. While the latter question for assessing commuting to school has not been formally validated, it is very similar to other 1-item questionnaire on children’s commuting to school that have demonstrated acceptable validity in this age group [59, 60]. The primary outcome will be categorical (“active” if children report that they walked or cycled to and/or from school, and “passive commuters” if children report that they traveled to and from school by car, motorcycle, bus, or train) and a second outcome will be continuous regarding the number of reported trips (max. Number of possible trips =10). This second outcome will then be categorized as active if they walk (reporting at least 4 walking trips) or cycle (reporting at least 3 cycling trips) [61]. Children will complete the questionnaire with the help of the teacher and researcher at school. The Spanish and English versions of the questionnaire are available at http://profith.ugr.es/pages/investigacion/recursos/paco.

#### Physical fitness

Physical fitness (cardiorespiratory fitness, muscular strength, and speed-agility) will be assessed using the ALPHA fitness test battery [62]. This fitness test battery consists of four tests: 20-m shuttle run test, handgrip strength, standing long jump test, and10-meter shuttle run test [62]. All children will complete the fitness test during a PE class. The same researchers will perform the fitness measurements at each school. Measurements will be organized in a circuit, and children will perform each test consecutively, except for the cardiorespiratory fitness test, where several participants will perform the measurements at the same time.

#### Anthropometry

Weight, height, and waist circumference will be assessed wearing PE clothes (shorts and a short sleeve shirt) and with the feet bare. Weight will be measured with a 0.1 kg approximation using a Seca 876 weighing system (Seca, Ltd., Hamburg, Germany). Height will be measured using the Frankfort plane, with a0.1 cm approximation using a Seca 2013 stadiometer (Seca, Ltd., Hamburg, Germany).Waist circumference will be assessed in a horizontal plane, at the level of the natural waist, by the measuring tape Seca 201(Seca, Ltd., Hamburg, Germany). Height, weight, and waist circumference will be measured twice, and the average of the two measurements will be used in the analysis. Body mass index (BMI) will be calculated as the weight in kilograms divided by the square of the height in meters. Weight status will be determined from BMI using age- and gender-specific cut points [63].

#### Sleep health

Assessment of sleep health will include both objective sleep parameters (total sleep time, sleep onset latency, sleep efficiency, and wake after sleep onset) and subjective parameters (sleep knowledge and hygiene, parents reported sleep symptoms, and sleep behaviors).

##### Objective sleep assessment

Objective sleep parameters will be assessed using an accelerometer (Actigraph wGT3X-BT, Pensacola, FL, USA).

##### Children sleep log

Children, in collaboration with their parents, will fill out a sleep log indicating the time they go to bed and the time they wake up to assess total sleep time.

##### Sleep knowledge and hygiene

Children’s sleep knowledge will be assessed using the Sleep Knowledge and Sleep Hygiene questionnaire developed by Philips as part of the educational program “I have a dream” (Spanish adaptation of the SimplyHealthy@Schools International Program; Philips Ibérica, S.A., Madrid, España). This questionnaire includes six questions about sleep knowledge and sleep-related behaviors (sleep hygiene and bed time routine).

##### Pediatric sleep questionnaire [64]

Parents will complete the questionnaire to report general children’s sleep behavior, symptoms related to sleep disorders (especially, sleep apnea, enuresis, and parasomnias), and daytime behavior (hypersomnolence, inattention, hyperactivity). The questionnaire contains 71 items with response categories “yes”, “no”, and “don’t know”, and 18 four-point Likert-type items. This questionnaire has been validated in Spanish children [65]*.*

##### Pediatric daytime sleepiness scale [66]

This questionnaire assesses daytime sleepiness-related behavior. The scale contains 8 items answered by parents and scored from 0 to 4, using a 5-point Likert-scale. The questionnaire was translated into Spanish and has been tested for comprehension in Spanish children [67].

#### Academic achievement

Academic achievement will be determined from the final grades in the official school records at the end of each trimester of the academic year in which data are collected. The academic indicators will be the grades (ranging from 0 to 10) from selected subjects (Natural Science, Social Sciences, Language (Spanish), Foreign language (English), Music, Arts, and PE) and the average grade point (sum of the grades obtained in each subject divided by the total number of subjects).

#### Health-related quality of life

Health-related quality of life will be assessed using the *Revidierter KINDer Lebensqualitätsfragebogen* (KINDL-R) [68], validated for Spanish children aged 4 to 16 years [69]. The KINDL-R consists of 24 items associated with 6 dimensions of health-related quality of life: physical well-being (e.g., illness, pain, fatigue), emotional well-being (e.g., boredom, loneliness, scared), self-esteem (e.g., pride, feeling on top of the world), family (e.g., relationship with parents, conflicts at home), friends (e.g., getting along with others or feeling different), everyday functioning in school (e.g., enjoying classes, worrying about the future), and disease (e.g., illness uncertainty, parent overprotection, missing school). Both children and parents will complete the respective versions of the KINDL-R questionnaires. The total score of both child and parental questionnaires will be transformed to a scale of 0 to 100, where higher scores indicate better health-related quality of life.

### Data analysis

Demographic characteristics will be analyzed using descriptive statistics. Data will be presented using the mean and standard deviation or median and interquartile range, where appropriate for continuous variables, and frequency and percentage for categorical variables. To assess the effectiveness of the different interventions on objectively measured PA and the other outcomes, the generalized linear model will be used with the outcome measures as dependent variables in separate models, the intervention as an independent variable and controlling for potential confounders (such as gender, body mass index, socioeconomic status, and baseline PA). Statistical significance will be set at *p* < 0.05.

## Discussion

The PREVIENE Project aims to determine the effectiveness of four school-based interventions (active commuting to/from school, active PE lessons, active school recess, sleep health promotion) implemented separately as well as simultaneously in primary school children. Outcome variables will include PA, fitness, anthropometry, sleep health, academic achievement, and health-related quality of life.

Although some interventions to promote PA in primary schools have been previously evaluated [1, 22–24, 70], no previous study has examined the simultaneous implementation of the multiple interventions included in the current study. Limited evidence suggests the effectiveness of multicomponent interventions to increase children overall daily PA through active commuting, PE lessons, and leisure time out school in The Netherlands [71, 72] and through recess time, PE lessons, in-class activities, and theme activities in Denmark [73]. In addition, only seven studies have examined the effects of school-based interventions to increase PA levels in children in Spain. None of such interventions had multiple components, and six of them focused on developing healthy lifestyle habits and one focused on PE lessons [74]. These interventions were effective to increase PA and the number of PE lessons during school time [75–77]. Therefore, the PREVIENE Project will advance the knowledge regarding the implementation and effectiveness of each of the five school-based interventions examined in this study. The results of this study will inform the design of future school-based interventions for increasing PA in children.

The present study has several strengths. The study interventions have been designed to minimize work imposed on teachers. They will focus on modifying current instructional strategies and using the existing resources. Therefore, the proposed interventions will be easy to implement at low cost. The teachers’ participation in the design and implementation of each of the interventions will allow them to gain the knowledge and skills required to train other teachers interested in using the methodology proposed by the PREVIENE Project. This will offer the participating teachers the opportunity to provide input on the design of study interventions to make them more feasible for implementation in school settings, and will facilitate development of the network of physical activity promoting schools in the city of Granada [78].

This study has several limitations, including a relatively small sample size and that teachers will implement all the intervention. The Research team will guide and control the correct application of each intervention program.

## Conclusion

The PREVIENE Project will examine the effectiveness of four simple and low-cost school-based interventions, implemented separately as well as simultaneously, on the PA level, fitness, anthropometry, sleep health, academic achievement, and health-related quality of life in primary school children. The main implication of developing these interventions is the possibility of increasing PA level in school children using programs that could be easily implemented in school settings. The development of a multicomponent intervention is crucial to change children’s PA habits. The results will extend the current knowledge about the effectiveness of different school-based interventions to increase PA level in primary school children.

## Abbreviations

BMI: Body Mass Index
MVPA: Moderate-to-vigorous physical activity
PA: Physical activity
PE: Physical Education
